# Application of Machine Learning for Mean Glandular Dose Prediction Utilizing DICOM Mammography Images

**DOI:** 10.3390/jimaging12070330

**Published:** 2026-07-21

**Authors:** Ali A. A. Alghamdi

**Affiliations:** Department of Radiological Sciences, College of Applied Medical Science, Imam Abdulrahman Bin Faisal University, P.O. Box 2435, Dammam 31441, Saudi Arabia; alalghamdi@iau.edu.sa

**Keywords:** mammography, mean glandular dose, artificial intelligence, machine learning deep learning, DICOM dosimetric data

## Abstract

The growing demand for raw and processed scientific data has encouraged many researchers and research institutions to adopt an open-source data policy. At present, data accessibility is of paramount importance due to the growing demand for artificial intelligence (AI) and machine learning (ML) applications in various scientific fields, particularly medicine. Medium- to large-scale mammography datasets are widely used in breast cancer research to develop and evaluate computer-aided detection methods. However, there are only a few studies on using mammogram datasets for the prediction of the breast mean glandular dose (*MGD*) with AI or ML models. The aim of this study was to investigate the feasibility of using ML and deep ML for *MGD* prediction based on DICOM images and retrieved dosimetric data from DICOM mammogram images. A total of 26,988 mammography images in DICOM format were obtained from the Federated Research Data Repository (FRDR). Eleven regression algorithms and three neural network-based models were evaluated using five-fold cross-validation. In addition, a deep ML fusion model based on Vision Transformer (ViT) and tabular data was developed for the prediction of the *MGD* normalized conversion factor CF(DgN). A mean breast thickness of 61.37 mm and a mean *MGD* of 1.53 mGy (0.55–6.33 mGy) were calculated using this dataset. Regarding tabular data, the artificial neural network (ANN) sequential models outperformed other linear and tree-based models. The ViT deep ML fusion model was tested with three configuration versions differing on the number of features included. A comparison of the three versions revealed that the version with six features achieved the best overall predictor performance. This study demonstrates that ML and deep ML can effectively predict the *MGD* using dosimetric tabular data and mammography DICOM images. The use of ML with tabular data extracted from DICOM images can be further strengthened by incorporating larger and more diverse datasets.

## 1. Introduction

The growing demand for raw and processed scientific data has encouraged many researchers and research institutions to adopt an open-source data policy. To some extent, this includes the use of software for processing and analyzing data directly related to or independently available for general purpose use. At present, data accessibility is of paramount importance due to the growing demand for artificial intelligence (AI) and machine learning (ML) applications in various scientific fields, particularly medicine. Open-source data policies have always led to better integration of ideas. Further, they effectively contribute to solving challenges and decreasing risks through facilitating learning. AI and ML can provide meaningful and accurate predictions if the dataset used is large enough to reflect the patterns in the data. Andresz et al. [1] have highlighted that constructing a dataset is the first step in the process of AI or ML model building. According to Barlas and Heavey [2], further processes to prepare a dataset for different evaluation stages may encompass several scientific branches, including computing, coding, data visualizations, data engineering, data sciences, and specific field expertise.

Mammography screening procedures remain the modality of choice for early breast cancer detection. However, several factors may contribute to increasing the radiation dose to the breast, especially to radiosensitive tissues like glandular tissues, leading to a higher risk of induced cancer. Continuous assessment and monitoring of radiation dose is therefore essential for establishing the limits and refining the mammography exposure parameters [3,4]. In breast cancer research, medium- to large-scale mammography datasets are widely used to develop and evaluate computer-aided detection methods. The publicly available Digital Database for Screening Mammography, developed at the University of South Florida, contains more than 2600 mammography studies and is a benchmark resource for training AI and ML models for breast cancer detection [5,6]. The U.S. National Mammography Database contains 3,181,437 collected and analyzed screening mammograms performed between January 2008 and December 2012 [7]. In the United Kingdom, the OPTIMAM (2008–2013) and OPTIMAM2 (2013–2018) projects assembled large image repositories to evaluate factors influencing breast cancer detection. The database comprises 3,072,878 images, with the number continuing to grow through ongoing projects [8]. More recently, the INbreast dataset, a publicly available collection of digital mammograms with ground-truth annotations for masses, calcifications, and other abnormalities, has become a key resource [9]. A convolutional neural network has been trained on 7632 mammography images from this dataset for breast cancer classification. Additional publicly available datasets support imaging and diagnostic research in other modalities, including ultrasonography [10], magnetic resonance imaging [11], and histopathological examination [12]. A recent review by Kendall et al. [13] summarized the range of currently accessible breast mammography image datasets.

There are only a few studies on the use of mammogram datasets to predict the breast *MGD* using AI or ML models. Massera and Tomal [14] introduced a framework that uses homogeneous breast models and artificial neural networks (ANNs). The data on dose assessments were generated using Monte Carlo (MC) simulations [15], and the study included 208 homogeneous virtual phantoms. Another framework that included 126 breast phantoms based on real heterogeneous computed tomography (CT) scan images obtained from real patients was developed by Saron et al. [16]; the dataset, which includes 60 digitally compressed phantoms, is publicly available. Sarno et al. [17] have recommended the use of tabular data and MC dose prediction for *MGD* and parametric phantom specification. The use of ML regression models reduces the requirements for computational capacity. Ma et al. [18] showed an association between the normalized average glandular dose and improved classification of breast cancer using retrospective data. In their study, logistic regression analysis was performed to establish the breast cancer risk assessment model. The sample included 1867 participants, and the average glandular dose in each mammogram view was extracted from the DICOM headers. Only the extracted tabular data, not DICOM images, were used directly. The authors concluded that the differences in the normalized average glandular dose between breasts with cancer and healthy breasts could be an independent risk factor. Collectively, these studies indicate the viability of ML-based dose prediction but are limited by small data, use of simulated phantoms, or the omission of imaging data.

The current study aims to address these limitations by exploring the possibility of forecasting *MGD* with the help of AI and ML models that were trained on open-source and real-world full-field digital mammography data. The dosimetric parameters obtained directly through DICOM headers were compared with physics-based *MGD* computations and processed on tabular ML models and a multimodal deep learning fusion architecture, with which mammographic images were incorporated. The publicly available Newfoundland and Labrador Breast Screening (2025) (NL-Breast-Screen) dataset was used to test model performance, generalization, and the possibility of using open-source mammography data to assess AI-driven dosimetric measurement.

## 2. Materials and Methods

An empirical, retrospective analytical approach was used in this study to approximate *MGD* in the entire field of digital mammography using a combination of dosimetric calculations of physics with machine and deep learning algorithms. Tabular ML and multimodal deep learning fusion architecture were used to extract, process, analyze, and model structured dosimetric parameters obtained via DICOM headers and mammographic images to test the predictive performance and generalization. A total of 26,988 Full-Field Digital Mammography (FFDM) images in DICOM format were obtained from the Federated Research Data Repository (FRDR) breast screening program, encompassing approximately 350 GB of data. The data were collected with Senograph Essential digital mammography machines (General Electric, Chicago, IL, USA) between 2008 and 2010 [19]. The images were organized into three primary folders labeled positive, normal, and false positive. Comprehensive dataset details are provided in the work of Kendall et al. [13]. A corresponding metadata file is included but does not currently contain the dosimetric information required for this study. The methodology comprised the following steps: (i) extraction of relevant dosimetric parameters, (ii) *MGD* calculation, and (iii) tabular and image data preparation for ML and ANN model analysis and fitting.

### 2.1. Preparation of Dosimetric Metadata

Digital mammography DICOM images typically contain embedded dosimetric parameters, including but not limited to the kilovoltage peak (kVp), patient age, source-to-patient distance, exposure time, X-ray tube current (mA), filter type, anode target material, breast thickness, view position, filter material, relative X-ray exposure, source-to-entrance distance, and entrance dose (mGy). These parameters were extracted directly from the DICOM headers using a Python 3.12.11 script, which was also employed for all subsequent data-processing steps in this study. The first step involved creating a Pandas DataFrame column containing the file path to each mammogram and exporting it as a comma-separated values (CSV) file. Next, dosimetric parameters were retrieved by accessing the relevant DICOM tags for each image using the stored paths, and each parameter was appended to the same CSV file. These procedures were repeated for all dataset folders to generate the initial merged dosimetric-metadata draft. For calculation efficiency, the anode target and filter materials were combined into a single column (anode–filter material) using elemental abbreviations (Mo/Mo, Rh/Rh, Mo/Rh, and W/Rh).

The *MGD* was estimated using the following formula:

(1) MGD=K.g.c.s,

where *K* represents the entrance surface air kerma (ESAK) without backscatter, as reported in the DICOM headers, and *g*, *c*, and *s* are conversion factors derived from the work of [20,21]. A Python script linked the extracted metadata kVp, anode/filter materials, breast thickness, and entrance dose to interpolated conversion factors from the tables published by [20,21] to calculate the breast tissue glandularity (*g*), anode/filter factor (*c*), and X-ray spectral correction factor (*s*). The *MGD* was also normalized to the entrance dose to yield the conversion factor CF(DgN). Normalization of *MGD* with the entrance dose to obtain CF(DgN) minimizes scale dominance and allows the deep learning models that accommodate image-based features to converge stably. Image dimensions were appended to the dataset to support future image inclusion and size standardization. The final dosimetric metadata were stored as a CSV file comprising 13 columns and 26,984 rows. A few images (<0.1%) were excluded as they lacked or had invalid dosimetric DICOM tags, leading to slight changes in figure in further preprocessing stages.

### 2.2. Data Preparation for ML

ML and deep learning rely on structured pipelines for deployment and interpretation. These procedures comprise preprocessing, feature selection, model selection, model evaluation and optimization, deployment, and subsequent monitoring and refinement [22]. A supervised ML approach was adopted to predict the *MGD* from the tabular data. Dosimetric metadata was partially pre-processed as described in Section 2.1. The resulting CSV file was imported into a Pandas DataFrame in Python, and feature selection was performed by dropping non-relevant columns. In addition to using the *MGD* as the prediction target, after excluding three features (the file path, relative exposure, and image size), the retained predictors included the kVp, exposure time, X-ray tube current, breast thickness, entrance dose, anode/filter materials, age, and projection view. Five of these variables were already numeric. The two categorical variables (anode/filter material and projection view) were converted to numeric form with the LabelEncoder function, after which the original categorical columns were dropped. Tabular ML models were encoded using label encoding because tree-based and neural network regressors are not sensitized to ordinal assumptions. Deep ML fusion models in turn represented categorical variables using one-hot encoding to prevent the effects of spurious ordering. The final dataset comprised 26,974 rows × 8 feature columns, with 12 rows removed because of missing or invalid values. Age had roughly 1000 entries missing, although some ML models can handle missing data. To reduce distortion of distribution, median imputation was chosen, and missing age values were imputed with the median value of 59. The impact of age on model performance was then assessed using feature importance analysis and exclusion experiments. Further details of age distribution analysis can be found in Kendall et al. [13].

### 2.3. Model Selection and Evaluation

#### 2.3.1. Tabular Data ML

The prepared dataset was analyzed using a correlation matrix. Relationships between each feature and the target variable (*MGD*), as well as their linear trends, were examined, aiding in the selection of an appropriate model type. Only training data in each cross-validation fold were scaled with feature scaling, and then the same procedure was applied to the respective validation/test sets to avoid data leakage. Eleven regression algorithms were initially evaluated with default parameter settings: LinearRegression (LR), least absolute shrinkage and selection operator (LASSO), and ElasticNet (EN) from sklearn.linear_model; DecisionTreeRegressor (CART) from sklearn.tree; KNeighborsRegressor (KNN) from sklearn.neighbors; SupportVectorRegressor (SVR) from sklearn.svm; RandomForestRegressor (RFR), GradientBoostingRegressor (GBR), ExtraTreesRegressor (ETR), and AdaBoostRegressor (ABR) from sklearn.ensemble; and eXtreme Gradient Boosting (XGB) from xgboost (scikit-learn library v1.7.1). Three neural network-based models were evaluated for comparison: MLPRegressor (MLP) from sklearn.neural_network, a TensorFlow Sequential (v2.20.0) model (tensorflow.keras.models; TF-Seq), and a PyTorch model (v 2.12.0) using torch.nn integrated with NeuralNetRegressor (Torch-Seq). The default scikit-learn MLP employs a single hidden layer of 100 ReLU-activated neurons with the Adam optimizer. A simple feedforward architecture was implemented for TF-Seq and Torch-Seq: an input layer matching the number of features, followed by two fully connected hidden layers (64 and 32 neurons and ReLU activation) and a single-neuron output layer with linear activation. Both models were compiled with the Adam optimizer. The dataset was randomly split into training (75%) and testing (25%) sets. Five-fold cross-validation with random shuffling was performed for all models during hyperparameter optimization. Model performance was assessed using mean squared error (MSE) and the coefficient of determination (R^2^) on both training and testing sets. The random seed was fixed at 44, and the number of estimators (n_estimators) was set to 400 where applicable. All hyperparameters, except the number of estimators (400 in the case of the ensemble models), were set as default to provide reasonable model comparisons and prevent overtuned predictive control.

#### 2.3.2. Tabular and Image Data Deep ML Fusion Model

Vision Transformer (ViT) enables a different approach to the field of visual learning through the use of a self-attention mechanism. This method was first created for language processing in the transformer architecture [23] and was then applied directly to image data [24,25]. The conventional method relies on convolutional neural networks (CNNs). The CNN method uses biases, kernel operations, and layered feature gathering [26,27]. ViT handles an image as a sequence of linearly embedded fixed size patches and processes the resulting token via a stack of multi-head self-attention with learned positional encodings of feed-forward layers [23,24]. ViT offers greater architectural flexibility and scalability, making it particularly suitable for multimodal fusion tasks. Details of the core model theory and basic structure have been covered in many recent studies; for example, see [23,24,25].

**Figure 1:**
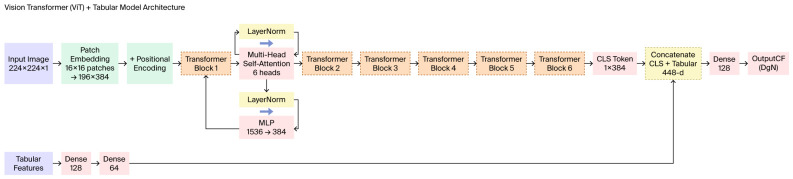
Deep ML fusion model architecture for tabular data and images.

Figure 1 illustrates a flowchart of the model architecture employed to process both tabular and image data for the prediction of the *MGD* normalized conversion factor CF(DgN). The CF(DgN) value normalized to the entrance dose is more suitable for deep ML with image data. Categorical data were encoded using one-hot encoding rather than label encoding to ensure appropriate feature representation for deep ML.

The model architecture has two branches. The image branch uses a ViT backbone that works on channel 224 × 224 DICOM inputs. The image is split into fixed-size non-overlapping patches of 16 × 16 pixels, which gives 196 patches. The patches are linearly projected to a 384-dimensional embedding space using a patch-embedding convolution. The tabular branch processes the tabular data. The image branch and the tabular branch merge to produce the regression output. A 384-dimensional positional embedding was added to preserve spatial ordering. The embedded sequence then passes through six transformer encoder blocks. The encoder is made up of six transformer blocks, with each block having a multi-head self-attention module with six attention heads followed by a feed-forward MLP. Each block has independent parameters; the architecture does not form a single 36-head attention layer. Each attenuation head uses a key/query/value with dimension 64. After the head self-attention layer, the block adds a feed-forward MLP, with an inner dimension of 1536 and GELU activation. Finally, the block applies a layer normalization layer, and the same process is repeated for each block. The encoder uses six heads and six blocks. The final ViT representation comes from the transformed [CLS] token, which holds the visual context of the image. The tabular branch takes in numeric and one-hot-encoded categorical variables and has a layer of 128 units and ReLU activation with a dropout of 0.3, followed by a dense layer of 64 units. The output of the ViT [CLS] token (dimension 384) and the tabular embedding (dimension 64) are concatenated to produce a fused representation with dimension 448. The combined vector passes through a connected layer with 128 units and ReLU activation and then passes through a dropout stage with a rate of 0.3. After the dropout stage, the combined vector reaches a linear output neuron that performs the final regression. The model applies dropout for regularization inside the transformer MLP blocks with a rate of 0.1 and inside the tabular and fusion layers with a rate of 0.3. The training uses the Adam optimizer, with a learning rate of 1 × 10^−4^ with MSE loss and the MAE metric, and the validation uses stopping and best checkpoint saving. This configuration allows the model to jointly learn high-level image features and structured tabular data. A five-fold cross-validation analysis with random shuffling was applied across all model configurations. The computer used to run the deep ML fusion model was a System76 Oryx Pro, equipped with 96 GiB of RAM and an AMD Ryzen AI 9 HX 370 w/Radeon 890M processor running Pops!_OS 24.04 LTS.

## 3. Results

### 3.1. Calculation of MGD

**Figure 2:**
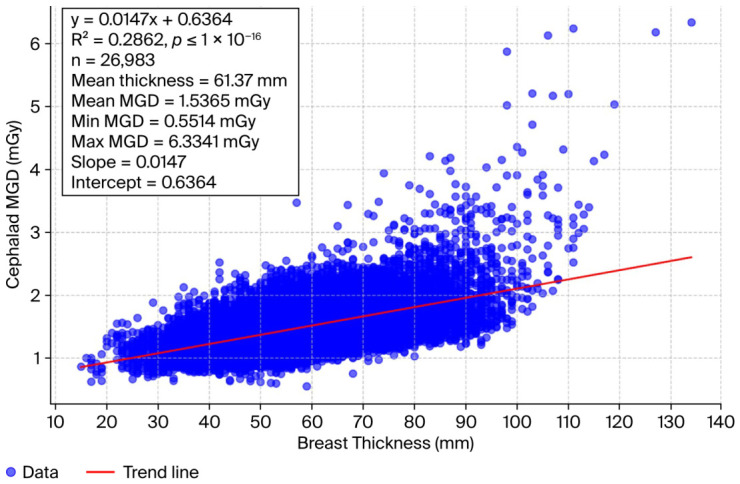
*MGD* (mGy) vs. breast thickness (mm).

Figure 2 shows the calculation of the *MGD* using Equation (1) plotted against breast thickness. In this dataset, a mean breast thickness of 61.37 mm was obtained. The mean *MGD* was 1.53 mGy, with a minimum of 0.55 mGy and a maximum of 6.33 mGy (*p* < 1 × 10^−16^, R^2^ = 0.2862). Variation in the *MGD* with breast thickness reflects the relationship described in Equation (1), representing the dose absorbed by glandular breast tissue. As expected from mammography physics, thicker breasts require higher radiation doses to compensate for increased X-ray attenuation, resulting in higher *MGD* values. The moderate R^2^ (0.2862) combined with the high statistical significance (*p* < 1 × 10^−16^) indicates that breast thickness is an important predictor but not the sole determinant of the *MGD*.

### 3.2. Feature Correlation Analysis

**Figure 3:**
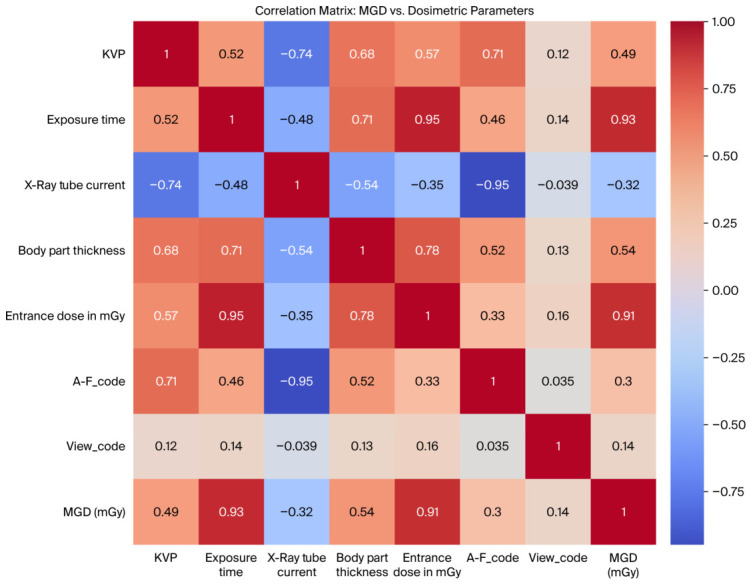
Correlation matrix: *MGD* vs. dosimetric parameters.

Figure 3 presents the correlation matrix of the eight selected features with the target variable *MGD*. The exposure time (0.93) and entrance dose (0.91) showed very strong positive correlations with the *MGD*. Breast thickness (0.54) and kVp (0.49) exhibited moderate positive correlations, whereas anode/filter code (A-F_code; 0.30) and projection view code (View_code; 0.14) had weak positive correlations. Age (−0.016) showed a low negative correlation, while the X-ray tube current (−0.32) showed a moderate negative correlation. Despite the negative correlation, the X-ray tube current was retained because of its physical relevance. When the prediction target was changed to CF(DgN), this feature demonstrated the highest absolute correlation (0.49), with all correlations being negative. Feature importance analysis using the feature_importances_ attribute of the RFR and XGB models ranked the X-ray tube current fifth, with importance scores of 0.009554 and 0.012548, respectively, ahead of age, A-F_code and View_code features.

### 3.3. Tabular ML Models

**Figure 4:**
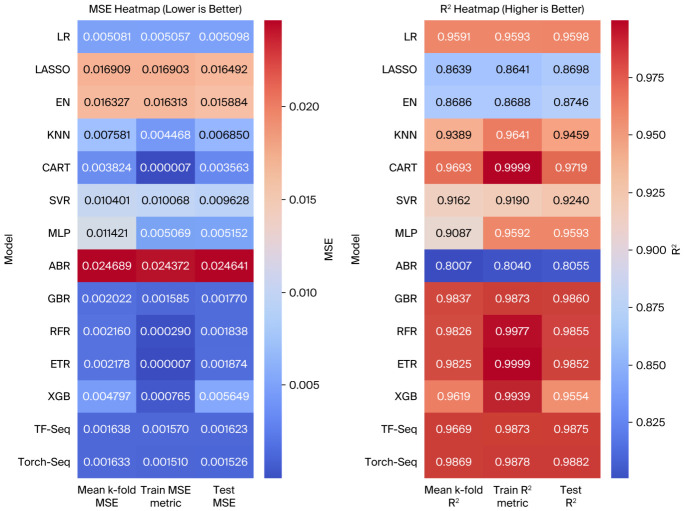
Heatmap showing the mean *k*-fold training and testing MSE and R^2^ values across all models.

Figure 4 presents a heatmap of the mean k-fold cross-validation metrics (training and testing MSE and R^2^) for all models. Torch-Seq and TF-Seq showed minimal differences between training and testing MSE, and their testing R^2^ values were slightly higher than their training values, indicating robust performance of the ANN-based models.

In contrast, the GBR, RFR, ETR, XGB, and CART methods exhibited wider gaps between training and testing MSE and R^2^, suggesting overfitting. The LR, LASSO, EN, SVR, MLP, KNN, and ABR approaches demonstrated consistent training and testing R^2^ values with minimal gaps, indicating limited overfitting; however, their lower mean k-fold R^2^ values reflected reduced predictive power compared with the top-performing models. The ANN-based models (Torch-Seq and TF-Seq) achieved the best overall performance, yielding the lowest mean k-fold MSE (0.00163) and the highest mean k-fold R^2^ (0.987), reflecting their capacity to capture complex, nonlinear patterns. The tree-based ensemble models GBR, RFR, and ETR performed nearly as well (mean k-fold R^2^: 0.982–0.984), offering simpler implementation and robustness to feature scaling, but showed slight overfitting with small training–testing gaps in MSE and R^2^. Among these models, GBR marginally outperformed RFR and ETR, achieving a lower MSE and minimal overfitting. XGB also demonstrated strong predictive ability but exhibited more overfitting than the other ensembles. The linear models (LR, LASSO, and EN), KNN, SVR, and MLP underperformed relative to the ensembles and ANN models. CART showed clear overfitting, with the largest discrepancy between training and testing MSE and R^2^. ABR delivered the weakest results, with the highest mean k-fold MSE (0.024689) and the lowest mean k-fold R^2^ (0.8007), making CART and ABR the least suitable for this prediction task.

The ANN-based model outperformed other models, as demonstrated in the last results. Steps were taken to rule out the possibility of target leakage, i.e., the possibility that the model learned the relation of some features for *MGD* prediction through Equation (1). Some features show a direct relationship in the *MGD* calculation; specifically, the entrance dose is the main factor in Equation (1). This is followed by a smaller direct relation for other factors when interpolating the glandularity factor (g), including breast thickness, age, kVp, and anode/filter for factor (c). While it is not practical to remove all potential sources of leakage, additional data fitting was performed to test this possibility. The ANN-based models (Torch-Seq and TF-Seq) were employed for data fitting without the entrance dose and age features. Table 1 shows the mean k-fold cross-validation, training, and testing metric scores.

The Torch-Seq model scored slightly better than TF-Seq across all metrics shown in Table 1. Both models scored lower than the previous models with the seven selected features. However, both models generally performed excellently even when excluding entrance dose and age from the selected features.

### 3.4. Tabular and Image Deep ML Fusion Model

Three deep ML fusion model versions with different numbers of selected features were tested: version 1 includes eight features, version 2 excludes the entrance dose and age, and version 3 includes only two features, namely, breast thickness and position views.

Table 2 presents the final average metric scores for the five-fold cross-validation of the three model versions. Version 1 employs eight features, including entrance dose and age, and achieved a training MSE of 9.44 × 10^−4^ and validation MSE of 5.5 × 10^−5^ but showed a large drop of 94% in MSE between training and validation. This indicates potential overfitting or data leakage. Version 2, which excludes the entrance dose and age (six features), showed the best overall performance, with the lowest training MSE of 1.31 × 10^−4^ and the second-lowest validation MSE of 5.3 × 10^−5^. Version 3, employing only breast thickness and position views as its two features, showed a higher validation MSE and MAE of 8.9 × 10^−5^ and 5.69 × 10^−3^, respectively, indicating that the two features are important but not sufficient. Version 2 showed the overall best performance and demonstrated that feature analysis and selection could enhance model performance in deep ML fusion models.

**Figure 5:**
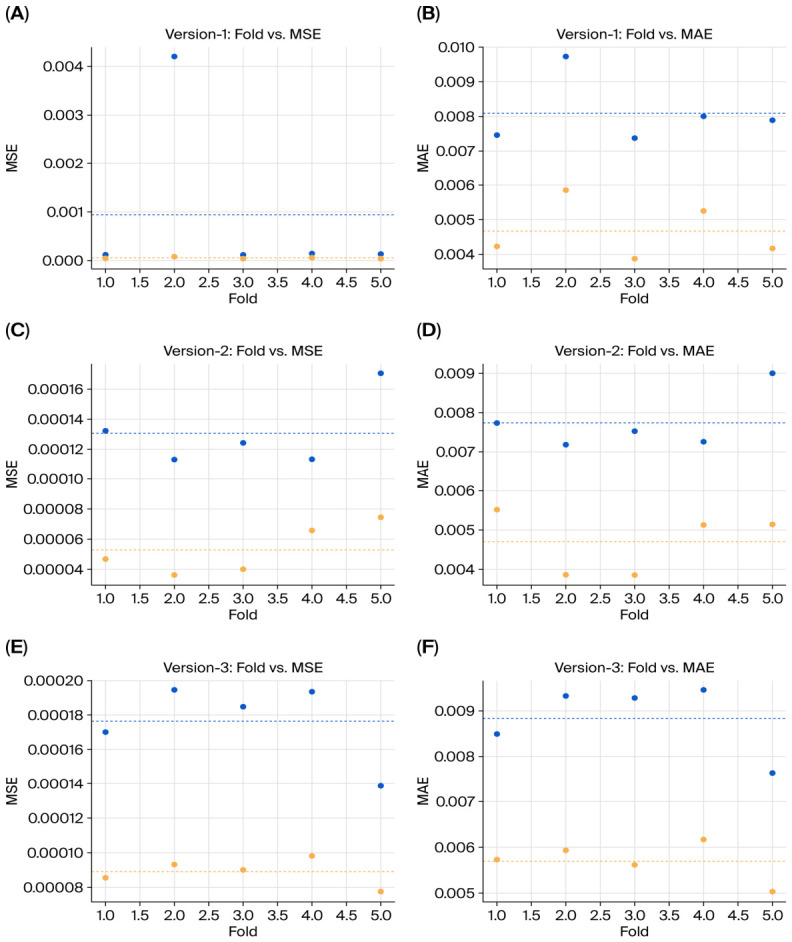
Cross-validation metrics MSE (**A**,**C**,**E**) and MAE (**B**,**D**,**F**) per fold/epoch score across model versions 1–3. The dashed lines indicate the mean values of each 5 k-fold for training (blue) and validation (yellow).

Figure 5 illustrates the training and validation metrics per fold across the three model versions. Figure 5A presents the training and validation MSE for model version 1. In the final epoch, fold 2 has a training MSE of 4.201 × 10^−3^, which is more than 30 times higher than the average for the other folds. This increases the mean training MSE to 9.44 × 10^−4^. The validation MSE stays low across all folds (4 to 8) × 10^−5^, with an average of 5.5 × 10^−5^. Figure 5B shows that the training MAE ranges from (7 to 9) × 10^−3^, and fold two is 25% higher than the average, reflecting the same effect seen in MSE and pointing to possible overfitting. The validation MAE is in the range of (3 to 5) × 10^−3^, showing strong generalization. However, the gap between training and validation (a 42% drop) is the largest among all versions, suggesting that the inclusion of the full feature set might pick up noise or data inconsistency during training that does not appear during validation. Figure 5C,D show the training and validation MSE and MAE for model version 2. The training MSE ranges from (1.13 to 1.71) × 10^−4^, with a mean value of 1.31 × 10^−4^. The validation MSE ranges from (3 to 7) × 10^−5^, with a mean value of 5.3 × 10^−5^. These values are the lowest and most consistent across all versions. The training MAE shows a range of (7 to 9) × 10^−3^, with the value for fold five being slightly higher than that of the other folds. The validation MAE shows a consistent range of (3.8 to 5.5) × 10^−3^ with a balanced training and validation gap (a 39% drop), which suggests good generalization without excessive signs of overfitting. Figure 5E,F show the training and validation MSE and MAE for model version 3. Using only breast thickness and position views (two features) results in the highest validation errors, with a validation MSE mean of 8.9 × 10^−5^ and a validation MAE of 5.69 × 10^−3^. However, the training and validation metrics are more consistent across folds than version 1. The training MAE is in the range of (7.6 to 9.4) × 10^−3^, with the value for fold five being lower than others, possibly due to easier data distribution in that split. The validation MAE stays in the range of (5 to 6) × 10^−3^, but the values are 20% higher than those obtained for versions 1 and 2. The gap between training and validation is much smaller (a 36% drop), which suggests less overfitting but also shows that the model has less capacity due to the inclusion of fewer features.

## 4. Discussion

### 4.1. MGD

The *MGD* values measured in the present study can be discussed in the context of the international dosimetry standards and recognized constraints of the methods of analytical dose estimation. The calculated average *MGD* indicates a compromise between the maintenance of diagnostic images and the observation of the existing radiation protection principles. Notably, most of the examinations have a lower level of diagnostic reference levels (DRLs) that are internationally acceptable, meaning that the imaging protocols adopted are usually optimized regarding managing the patient dose.

MC-based conversion factors, as suggested by UK, European, and IAEA guidelines, provide consistency in the methodology with the generally accepted dosimetry practices. For context, the European Commission considers 3 mGy an acceptable dose for a 50 mm compressed breast, with an achievable dose target of about 2.4 mGy (Figure 2) [28]. Regulatory agencies report similar reference limits: the U.S. Food and Drug Administration [29] specifies a maximum of 3 mGy per projection for a 42 mm breast with 50% fibroglandular tissue, the International Atomic Energy Agency (IAEA) [30] recommends 2.3 mGy for a 45 mm standard-composition breast, and the International Commission on Radiological Protection (ICRP) [31] sets a diagnostic reference level of 3 mGy per projection. These conversion factors convert incident air kerma to absorbed glandular dose, with assumptions relating to breast composition, beam quality and geometry [20]. Although this method is considered the clinical and regulatory standard, it is a simplified view of the breast anatomy since it presupposes a homogenous glandular tissue distribution. According to Dance et al. [21], such simplification may result in systematic uncertainty, especially in cases where the breast has a dissimilar density distribution or atypical distribution of glandularity.

Other methods, including LIBRA-based patient-specific glandular composition segmentation, follow the latter approach to address this limitation by directly estimating patient-specific glandular composition based on mammographic imaging. The concordance between LIBRA-derived and Dance-based *MGD* estimates reported by Borzì et al. [32] indicates that analysis techniques do not decline at the population level, even though accuracy at the individual level could be enhanced by the personalization of images. In the same fashion, MC-based models constructed by Sobol and Wu [33] and Boone [34] prove that various dosimetric models converge within reasonable error margins, as confirmed by comparative studies like those conducted by Suleiman et al. [35]. This agreement promotes the further operation of analytical *MGD* models in standard clinical practice, especially in cases where a complete MC simulation is not feasible.

Nevertheless, although full MC simulations are the best estimators, their computationally expensive nature and the need to have detailed volumetric breast phantoms restrict their application to large-scale screening programs [17]. The method of analysis employed in this research is thus a sensible trade-off: it allowed for prompt and standardized dose estimation, and it did not conflict with regulatory rules. Therefore, the reported values of *MGD* in this case were the estimated and not precise patient-specific absorbed doses but are considered to be clinically significant.

### 4.2. ML Model Performances and Applications

#### 4.2.1. Tabular ML Model Performances

The use of ML models for tabular dosimetric data points contributes to the growing use of data-driven techniques in medical physics, especially in scenarios where many interdependent parameters affect the result. In contrast to conventional regression models that presuppose linear or almost linear correlations, ML models can deal with nonlinear interactions among mammography acquisition parameters. The better results of ANN-based models in this research indicate that they can capture these complicated relationships. ANNs can be used to model higher-order relationships between exposure settings, beam quality, and anatomic factors that are difficult to model in linear models due to the use of a multilayer architecture and nonlinear activation functions. This observation is not new and aligns with the literature on ML-based dose estimation systems, which has demonstrated neural networks to be better than traditional regression models in their ability to predict radiological dose scales [36].

Nevertheless, ensemble tree-based models, such as RFR, GBR, and XGB, have demonstrated strong performance, indicating that both interpretability and performance should be considered when selecting a model. These tree-based models have benefits in clinical studies because they are comparatively robust to feature scaling, are partially resistant to missing data, and rank features by their importance [37]. This interpretability proves particularly useful in areas such as quality assurance and protocol optimization, where an explanation of the reasons behind prediction variation is as important as the prediction itself.

The tabular ML method has operational benefits for clinical implementation. DICOM header dosimetric parameters are easily accessible, can be obtained with little preprocessing, and can be calculated with limited computing costs. This renders tabular ML models appealing as part of standard quality control processes, and they could be employed to call out tests outside of the anticipated dose ranges or to compare performance across imaging systems. Furthermore, a logical and clinically important extension of these models to classification problems, such as the detection of examinations that are above regulatory limits, makes sense.

#### 4.2.2. Deep ML Fusion Model

A more ambitious model is the deep ML fusion model, which fuses structured dosimetric data with unstructured image information. Notably, the image branch uses a Vision Transformer (ViT) backbone. ViTs are effective in medical imaging tasks, including long-range spatial dependencies, providing an alternative to conventional convolutional neural networks (CNNs). The results of the assessment of various versions of fusion models can give valuable information regarding the connection between the choice of features, the complexity of the model, and the generalization. The performance of version 1 shows a typical difficulty of medical ML, namely, that the incorporation of features that are highly predictive but physically connected (e.g., entrance dose) causes artificially high validation performance to hide overfitting or data leakages. The significant difference between training and validation errors in this version highlights the importance of the need to reconcile the ML design decisions with the physics of the target variable.

Likewise, the findings with version 2 show that robustness could be enhanced by removing features that may confound the predictive power. Limiting analytical *MGD* calculation to entrance dose eliminates the chance of circular learning and the limitation of age to age-specific imbalance without excluding its clinical relevance in the dataset. This supports a significant, relevant, and context-dependent methodological concept that should be measured considering data quality and completeness limitations.

The findings obtained using version 3 further highlight that though breast thickness and projection view are the key determinants of *MGD*, they do not provide the full complexity of dose formation when used independently. The lower overfitting of this minimal feature model implies better generalization but with lower accuracy. The trade-off supports the purpose of feature selection strategies that are balanced in terms of physical relevance and statistical strength. Consequently, the fusion model illustrates that it is possible to combine mammographic images with tabular dosimetric data. Although tabular features represent intent to acquire and system settings, spatial dose distribution and tissue composition are implicitly represented by the image branch. These hybrid methods can eventually allow for a more personalized estimation of the dose when model complexity is controlled.

### 4.3. Implications

This study has several significant implications for mammography dosimetry, clinical practice, and the design of ML-aid quality assurance instrumentations. Together, the findings indicate that the *MGD* behavior is regulated by multifaceted interactions between anatomical parameters and acquisition parameters, and these multifaceted interactions can be successfully approximated using well-constructed ML and deep learning models without breaking the rules of physical dose calculation.

First, on the clinical front, the capacity to accurately approximate the *MGD* based on DICOM-derived parameters, which can be readily obtained on a routine basis, has direct implication for the protection of patients from radiation [38]. The findings indicate that ML-based models can aid in continuous dose monitoring in examinations that use a dose outside the anticipated dose ranges, despite the lack or partiality of explicit dosimetric computations. This capacity is especially useful in scenarios where throughput screening is of high intensity and real-time feedback may assist radiographers in modifying the acquisition parameters to prevent an accidental dose increase while still achieving diagnostic image quality. Furthermore, the sensitivity of the *MGD* to parameter combinations but not to individual parameters used in an isolated setting is one of the reasons why protocol optimization strategies with consideration of multivariate interactions are needed. ML decision-support systems may help clinicians to implement exposure settings specific to individual patients, thus moving the idea of personalized radiation dose management forward.

Second, technically, the high performance of tabular ML models suggests that complex dose prediction is not always intensive in terms of computation requirements when dealing with images. The tabular dosimetric trained models can be implemented on common clinical devices and thus can be integrated into an existing radiology information system or image archiving and communication system (PACS). It is further hypothesized that image-based information can be used in fusion models to potentially supplement dose prediction in the presence of structured data, though only in cases where feature selection is well controlled. In the design of ML systems, a major consequence is that generalization is likely to be compromised with the inclusion of all possible features, but physics-informed feature selection can enhance the system’s resilience and comprehensibility. These results endorse a hybrid modeling approach where domain knowledge drives ML architecture and feature engineering.

Thirdly, methodologically, the study supports the necessity of designing ML models in line with the physics of the variable of interest. The performance degradation observed in the case where the entrance dose was not removed but increased provides evidence that ML models can be used to infer latent physical relationships as opposed to being an analytical calculation method. The generalization of this discovery in the use of ML in medical physics, in which a significant number of variables of interest are obtained as solutions of deterministic equations, is evident. The findings also underscore the importance of strident analysis of features, cross-validation, and sensitivity testing to make sure that predictive performance is indicative of actual learning and not data leakage or overfitting. The practices must be viewed as critical in the creation of ML tools for use in clinical decision support.

### 4.4. Limitations and Future Directions

Further evaluations are needed to rule out possible leakages, including sensitivity testing at patient and examination levels. Several additional constraints should be considered when interpreting the findings of this study. First, age data are not fully provided, and some analyses become incomplete without excluding features, thus restricting the inferences regarding the impacts of demographic factors on dose. Even though this choice enhanced the stability of the model using the current dataset, age is a clinically relevant variable that must be included in future research with complete datasets. Second, the analytical calculation of *MGD* is known to exhibit certain limitations related to homogeneous tissue assumptions and reduced breast geometry. Although these limitations are clearly acknowledged in the literature, they still act as a source of uncertainty in large-scale mammography dosimetry experiments.

Third, the sample is representative of a relatively small age range, which could limit the generalizability of the findings. Multi-center and multi-vendor datasets are thus necessary to provide external validation. Lastly, the deep fusion model creates great computational requirements. The ViT input requirements of high-resolution DICOM images can be fulfilled by resizing the images, but this can result in the loss of fine structural information, the effects of which should be studied further. It might be necessary to tackle this problem by employing higher-resolution architecture or patch-based learning. The next phase of the research would be to determine the external validity of the proposed models in different populations using different imaging systems. The use of volumetric breast density or direct glandularity measurements may be beneficial to increasing the physiological interpretability and refining the estimation of patient dose. More elaborate fusion schemes, such as the use of multi-scale image representations and weighting tabular features based on attenuation, could further enhance performance as well.

From a quality assurance perspective, the ML-based *MGD* prediction models can be used to supplement the current regulatory framework with automated benchmarking based on national and international diagnostic reference levels (DRLs). Future applications may include excess DRL detection, outlier dose detection, and analyses of the false negative rate, Bland–Altman agreement, and acceptable error range. Dose optimization and justification in medical imaging are gaining considerable importance in regulatory agencies. The methodologies used in this study accommodate such objectives by providing dose evaluation tools that can be scaled and applied in conjunction with standard guidelines from agencies like IAEA, ICRP, and FDA. Notably, the explicit assessment of target leakage is believed to enhance the physical usefulness of ML-assisted dosimetry, an issue that often raises concerns among regulators and clinical physicists.

Lastly, future clinical trials are required to determine the integration of ML-assisted *MGD* prediction into clinical mammography routines to enhance dose optimization, quality control, and regulatory adherence.

## 5. Conclusions

The study demonstrates the usefulness of open-source DICOM mammography datasets in the development of an ML-based *MGD* estimation. The results indicate that ANN-based models are generally better than traditional linear and tree-based models in terms of predictive accuracy, low cross-validation error, and high generalization, while also having low overfitting susceptibility. These findings suggest that nonlinear learning architectures are especially well placed to describe the complicated interactions of mammography acquisition parameters and patient-specific effects that determine mammographic dose. In addition to tabular modeling, the paper presents and comprehensively discusses a deep learning fusion framework that combines mammographic DICOM images and structured dosimetric data. The findings indicate the value of physics-based feature selection in hybrid architecture, and show that eliminating features that are more likely to undergo data leakage enhances the robustness of the model without affecting the predictive quality. The efficiency of the implemented Vision Transformer-based fusion model also demonstrates the possibility of integrating image-obtained data with tabular data to predict doses, and the performance of the model trades off between its complexity, generalization, and computational consumption. Overall, this research provides a sound methodology for applying ML and deep learning to mammography dosimetry, focusing on transparency, validation, and consistency relative to known physical dose models.

## Figures and Tables

**Table 1:** Torch-Seq and TF-Seq models score metrics without entrance dose and age features.

Model	K-Fold MSE	Training MSE	Test-MSE	K-Fold R^2^	Training R^2^	Test-R^2^
Torch-Seq	0.00352	0.00344	0.00359	0.971	0.972	0.970
TF-Seq	0.00367	0.00375	0.00385	0.970	0.970	0.967

**Table 2:** Final average metrics of 5 k-fold cross-validation for fusion deep ML model versions.

Model	Training MSE	Validation MSE	Training MAE	Validation MAE
Version 1	9.44 × 10^−4^	5.5 × 10^−5^	8.09 × 10^−3^	4.67 × 10^−3^
Version 2	1.31 × 10^−4^	5.3 × 10^−5^	7.74 × 10^−3^	4.70 × 10^−3^
Version 3	1.76 × 10^−4^	8.9 × 10^−5^	8.84 × 10^−3^	5.69 × 10^−3^

## Data Availability

The data presented in this study are available in FRDR Newfoundland and Labrador Breast Screening (NL-Breast-Screen) Dataset at https://www.frdr-dfdr.ca/repo/dataset/c702145e-2e53-4421-b578-8bb53bbd3952 (accessed on 1 August 2025). The data related to this work (dosimetric metadata file and Python codes) can be made available on reasonable request.

## References

[1] Andresz S., Zéphir A., Bez J., Karst M., Danieli J. (2022). Artificial intelligence and radiation protection. A game changer or an update?. Radioprotection.

[2] Barlas P., Lanning I., Heavey C. (2015). A survey of open source data science tools. Int. J. Intell. Comput. Cybern..

[3] Loveland J., Young K.C., Oduko J.M., Mackenzie A. (2022). Radiation doses in the United Kingdom breast screening programmes 2016–2019. Br. J. Radiol..

[4] Di Maria S., Vedantham S., Vaz P. (2022). X-ray dosimetry in breast cancer screening: 2D and 3D mammography. Eur. J. Radiol..

[5] Bowyer K., Kopans D., Kegelmeyer W.P., Moore R., Sallam M., Chang K., Woods K. (1996). The digital database for screening mammography. Third International Workshop on Digital Mammography.

[6] Heath M., Bowyer K., Kopans D., Kegelmeyer P., Moore R., Chang K., Munishkumaran S. (1998). Current status of the digital database for screening mammography. Digital Mammography: Nijmegen.

[7] Lee C.S., Bhargavan-Chatfield M., Burnside E.S., Nagy P., Sickles E.A. (2016). The national mammography database: Preliminary data. Am. J. Roentgenol..

[8] Halling-Brown M.D., Warren L.M., Ward D., Lewis E., Mackenzie A., Wallis M.G., Wilkinson L.S., Given-Wilson R.M., McAvinchey R., Young K.C. (2020). OPTIMAM mammography image database: A large-scale resource of mammography images and clinical data. Radiol. Artif. Intell..

[9] Huang M.L., Lin T.Y. (2020). Dataset of breast mammography images with masses. Data Brief.

[10] Al-Dhabyani W., Gomaa M., Khaled H., Fahmy A. (2020). Dataset of breast ultrasound images. Data Brief.

[11] Saha A., Harowicz M.R., Grimm L.J., Kim C.E., Ghate S.V., Walsh R., Mazurowski M.A. (2018). A machine learning approach to radiogenomics of breast cancer: A study of 922 subjects and 529 DCE-MRI features. Br. J. Cancer.

[12] Aksac A., Demetrick D.J., Ozyer T., Alhajj R. (2019). BreCaHAD: A dataset for breast cancer histopathological annotation and diagnosis. BMC Res. Notes.

[13] Kendall E., Hajishafiezahramini P., Hamilton M., Doyle G., Wadden N., Meruvia-Pastor O. (2024). Full field digital mammography dataset from a population screening program. arXiv.

[14] Massera R.T., Tomal A. (2020). Estimation of glandular dose in mammography based on artificial neural networks. Phys. Med. Biol..

[15] Massera R.T., Tomal A. (2021). Breast glandularity and mean glandular dose assessment using a deep learning framework: Virtual patients study. Phys. Med..

[16] Sarno A., Mettivier G., di Franco F., Varallo A., Bliznakova K., Hernandez A.M., Boone J.M., Russo P. (2021). Dataset of patient-derived digital breast phantoms for in silico studies in breast computed tomography, digital breast tomosynthesis, and digital mammography. Med. Phys..

[17] Sarno A., Massera R.T., Paternò G., Cardarelli P., Marshall N., Bosmans H., Bliznakova K. (2025). Uncertainty and normalized glandular dose evaluations in digital mammography and digital breast tomosynthesis with a machine learning methodology. Phys. Med..

[18] Ma L., Cai Y., Lin X., He Z., Zeng H., Chen W., Qin G. (2020). Association of the differences in average glandular dose with breast cancer risk. BioMed Res. Int..

[19] FRDR Newfoundland and Labrador Breast Screening (NL-Breast-Screen) Dataset. https://www.frdr-dfdr.ca/repo/dataset/c702145e-2e53-4421-b578-8bb53bbd3952.

[20] Dance D.R., Skinner C.L., Young K.C., Beckett J.R., Kotre C.J. (2000). Additional factors for the estimation of mean glandular breast dose using the UK mammography dosimetry protocol. Phys. Med. Biol..

[21] Dance D.R., Young K.C., Van Engen R.E. (2009). Further factors for the estimation of mean glandular dose using the United Kingdom, European and IAEA breast dosimetry protocols. Phys. Med. Biol..

[22] Hapke H., Nelson C. (2020). Building Machine Learning Pipelines.

[23] Vaswani A., Shazeer N., Parmar N., Uszkoreit J., Jones L., Gomez A.N., Kaiser Ł., Polosukhin I. (2017). Attention is all you need. Adv. Neural Inf. Process. Syst..

[24] Hassija V., Palanisamy B., Chatterjee A., Mandal A., Chakraborty D., Pandey A., Chalapati G.S., Kumar D. (2025). Transformers for vision: A survey on innovative methods for computer vision. IEEE Access.

[25] Utkin I.A., Shkuropatsky V.V., Pronikov A.N., Rakov E.S. (2024). The study of the vision transformer architecture by explainability methods. Inform. Telecommun. Control.

[26] Tolstikhin I.O., Houlsby N., Kolesnikov A., Beyer L., Zhai X., Unterthiner T., Yung J., Steiner A., Keysers D., Uszkoreit J. (2021). MLP-Mixer: An all-MLP architecture for vision. Adv. Neural Inf. Process. Syst..

[27] Raghu M., Unterthiner T., Kornblith S., Zhang C., Dosovitskiy A. (2021). Do vision transformers see like convolutional neural networks?. Adv. Neural Inf. Process. Syst..

[28] Perry N., Broeders M., de Wolf C., Törnberg S., Holland R., von Karsa L. (2008). European guidelines for quality assurance in breast cancer screening and diagnosis—Summary document. Oncol. Clin. Pract..

[29] U.S. Food and Drug Administration (FDA) Summary of Safety and Effectiveness. https://www.accessdata.fda.gov/cdrh_docs/pdf16/P160031B.pdf.

[30] International Atomic Energy Agency (IAEA) (2005). Optimization of the Radiological Protection of Patients: Image Quality and Dose in Mammography, IAEA-TECDOC-1447.

[31] International Commission on Radiological Protection (ICRP) Diagnostic Reference Levels in Medical Imaging (Supporting Guidance 2). https://www.icrp.org/docs/drl_for_web.pdf.

[32] Borzì G.R., Bonanno E., Cavalli N., D’Anna A., Pace M., Stella G., Zirone L., Marino C. (2025). Assessment of a patient dose monitoring system for average glandular dose (AGD) estimate in mammography. Appl. Sci..

[33] Sobol W.T., Wu X. (1997). Parametrization of mammography normalized average glandular dose tables. Med. Phys..

[34] Boone J.M. (2002). Normalized glandular dose (DgN) coefficients for arbitrary x-ray spectra in mammography: Computer-fit values of Monte Carlo derived data. Med. Phys..

[35] Suleiman M.E., Brennan P.C., McEntee M.F. (2017). Mean glandular dose in digital mammography: A dose calculation method comparison. J. Med. Imaging.

[36] Appelt A.L., Elhaminia B., Gooya A., Gilbert A., Nix M. (2022). Deep learning for radiotherapy outcome prediction using dose data—A review. Clin. Oncol..

[37] Weng Y., Fang Y., Yan H., Yang Y., Hong W. (2019). Bayesian non-parametric classification with tree-based feature transformation for NIPPV efficacy prediction in COPD patients. IEEE Access.

[38] Tsave O., Kosvyra A., Filos D.T., Fotopoulos D.T., Chouvarda I. (2025). A multi-dimensional framework for data quality assurance in cancer imaging repositories. Cancers.

